# Retention in care and factors critical for effectively implementing antiretroviral adherence clubs in a rural district in South Africa

**DOI:** 10.1002/jia2.25396

**Published:** 2019-10-06

**Authors:** Peter Bock, Colette Gunst, Leonard Maschilla, Rory Holtman, Nelis Grobbelaar, Dillon Wademan, Rory Dunbar, Geoffrey Fatti, James Kruger, Nathan Ford, Graeme Hoddinott, Sue‐Ann Meehan

**Affiliations:** ^1^ Desmond Tutu TB Centre Department of Paediatrics and Child Health Faculty of Medicine and Health Sciences Stellenbosch University Cape Town South Africa; ^2^ Department of Health Western Cape Government Cape Winelands District South Africa; ^3^ Division of Family Medicine and Primary Health Care Faculty of Medicine and Health Sciences Stellenbosch University Cape Town South Africa; ^4^ Department of Health Western Cape Government Cape Town South Africa; ^5^ ANOVA Health Institute Paarl South Africa; ^6^ Kheth’ Impilo AIDS Free Living Cape Town South Africa; ^7^ Division of Epidemiology and Biostatistics Department of Global Health Faculty of Medicine and Health Sciences Stellenbosch University Cape Town South Africa; ^8^ World Health Organization Geneva Switzerland

**Keywords:** HIV, antiretroviral treatment, differentiated care, adherence clubs, retention in care, lost to follow‐up, staff perceptions, clients’ perceptions, factors key for success

## Abstract

**Introduction:**

Differentiated models of care that include referral of antiretroviral treatment (ART) clients to adherence clubs are an important strategy to help clinics manage increased number of clients living with HIV in resource‐constrained settings. This study reported on (i) clinical outcomes among ART clients attending community‐based adherence clubs and (ii) experiences of adherence clubs and perceptions of factors key to successful adherence club implementation among clients and healthcare workers.

**Methods:**

A retrospective cohort analysis of routine data and a descriptive analysis of data collected through self‐administered surveys completed by clients and healthcare workers were completed. Clients starting ART at the study clinic, between January 2014 and December 2015, were included in the cohort analysis and followed up until December 2016. The survey data were collected from August to September 2017. The primary outcome for the cohort analysis was a comparison of loss to follow‐up (LTFU) between clients staying in clinic care and those referred to adherence clubs. Survey data reported on client experiences of and healthcare worker perceptions of adherence club care.

**Results:**

Cohort analysis reported on 465 participants, median baseline CD4 count 374 (IQR: 234 to 532) cells/μl and median follow‐up time 20.7 (IQR 14.1 to 27.7) months. Overall, 202 (43.4%) participants were referred to an adherence club. LTFU was lower in those attending an adherence club (aHR =0.25, 95% CI: 0.11 to 0.56). This finding was confirmed on analysis restricted to those eligible for adherence club referral (aHR =0.28, 95% CI: 0.12 to 0.65). Factors highlighted as associated with successful adherence club implementation included: (i) referral of stable clients to the club, (ii) an ideal club size of ≥20 members, (iii) club services led by a counsellor (iv) using churches or community halls as venues (v) effective communication between all parties, and (vi) timely delivery of prepacked medication.

**Conclusions:**

This study showed good clinical outcomes, positive patient experiences and healthcare worker perceptions of the adherence club model. Factors associated with successful adherence club implementation, highlighted in this study, can be used to guide implementers in the scale‐up of adherence club services across varied high‐burden settings.

## 1 Introduction

In 2015, the World Health Organization (WHO) changed antiretroviral treatment (ART) guidelines to recommend lifelong ART for all HIV‐positive individuals regardless of CD4 count 1. High burden countries, for example, South Africa, are now implementing this policy, which has led to an even greater number of HIV‐positive individuals starting ART at Primary Health Care (PHC) facilities, a trend associated with worsening clinical outcomes 2, 3, 4. For HIV treatment services, differentiated care is defined as *a client‐centred approach that simplifies and adapts HIV services across the cascade, in ways that both serve the needs of PLHIV better and reduce unnecessary burdens on the health system* 5. Referral of stable ART clients to adherence clubs as part of a differentiated care model is an important strategy for reducing clinic burden 2, 5, 6, 7, 8, 9.

Early studies have shown mixed results when implementing ART adherence clubs. A large study conducted in the Cape Metropole, South Africa, included 3216 clients and showed overall retention in ART care of 95% and 89% at 12 and 24 months respectively, after adherence club referral 10. Another study from Khayelitsha, Cape Town, showed higher rates of ART client retention over 18 months of follow‐up among 502 clients attending adherence clubs compared with 2327 clients not attending clubs (97% vs. 85%) 11, 12, 13. In contrast, a recent pragmatic trial conducted in Johannesburg, showed higher LTFU among ART clients in community‐based adherence clubs compared with clinic‐based adherence clubs. (HR 1.38, 95% CI 1.02–1.87) 14.

Perceived advantages of adherence clubs include improved access to services with reduced waiting times, social networking associated with being managed as a group 15, and an increased role for community healthcare workers (CHWs) 13, 16. However, healthcare workers have expressed concerns about lack of control of adherence club activities, particularly with respect to provision of ART and other chronic disease medications 7, 13, 15. This study reported on clinical outcomes among ART clients attending adherence clubs and client experiences and healthcare worker perceptions of factors key to successful adherence club implementation in the Cape Winelands District, South Africa.

## 2 Methods

### 2.1 Study setting

This study was conducted at one PHC clinic and three linked community‐based adherence clubs. The study clinic was included in a community‐randomized trial and implemented ART regardless of CD4 count for all HIV‐positive adults from 2014, prior to recent changes in ART guidelines 17. With this exception, all other aspects of ART care were provided according to local ART guidelines, which transitioned to ART regardless of CD4 count in October 2016 2, 8, 18. Clinic‐based ART services were nurse‐led 18, and included viral load testing at four and 12 months after ART initiation and annually thereafter. The first‐choice first‐line ART regimen was a fixed‐dose combination of tenofovir (TDF)/lamivudine (3TC) and efavirenz (EFV) 18.

Adherence clubs were introduced in the Cape Winelands in 2012. There were set criteria for adherence club referral (Table 1) 19. Eligible clients could choose to remain at the clinic or attend an adherence club 9, 19. If clients remained at the clinic they could receive their medication through the “fast lane” system which provided medication‐only collection visits. All clients, including those in both clinic and adherence club care, visited the clinic every six months for a clinical assessment, routine laboratory investigations and renewal of their prescription 18. Clients in adherence clubs could return to clinic care if they developed a clinical illness, for example, tuberculosis (TB) or if they became pregnant and were required to see a clinician at regular intervals 19.

**Table 1:** Simplified eligibility criteria for adherence club referral of stable ART clients

Inclusion criteria
Adult ≥18 years (adolescents could be seen in a support group/specific time as requested by them)
Must be on current ART regime ≥6 months. If there has been a single drug substitution, the clinician to determine when the patient is eligible for adherence clubs
Most recent (taken in past 6 months) viral load <400 copies/mL
ART adherence ≥90%
Patient agrees to receive care through the adherence club system
Exclusion criteria
Pregnant
Active tuberculosis

Taken from appendix C, Cape Winelands District Guideline for the distribution of pre‐packed medication to stable clients on chronic disease medication at “Fast‐Lane” or “Club” 19.

Clients attending adherence clubs were seen by nurses, counsellors and community healthcare workers (CHWs). When clients arrived at the clubs, their appointment cards were collected by a CHW. If the client reported feeling unwell, they were referred to the clinic on the same day, or, if not acutely ill, advised to make an appointment. Clients attended the clubs every two months and received two months pre‐packed medication, except for the period November to December, when they received a four‐month supply of medication, due to many people travelling over this holiday period. Additional services provided at the adherence clubs included provision of injectable contraception, condoms and health promotion material. Average duration of stay at the club was one hour. Common causes of delay in the visit time included delays in transport of medication from the clinic and staff absenteeism. Data on each clinic and adherence club visit were collected on the routine electronic monitoring system TIER.Net 20.

### 2.2 Study design

This study included both: (i) a retrospective cohort analysis of routine data from the study clinic and (ii) descriptive data collected through a self‐administered survey.

### 2.3 Cohort data, study design, data and analysis

Routine data were extracted from TIER.Net 20, National Health Laboratory (NHLS) and ETR.net data sets 21. TIER.Net was the primary data source. NHLS data were used to extract CD4 and VL results missing from TIER.Net. Data on TB treatment at time of ART start were extracted from ETR.net.

A retrospective cohort study of all clients ≥ 18 years starting ART between 1 January 2014 and end December 2015 at the study clinic and entered in TIER.Net, including pregnant women, was completed. Baseline was defined as “at the time of starting ART.” All clients were followed up until the date of death, LTFU, transfer out (TFO) or until end December 2016. Where available, data definitions used in TIER.Net were used. LTFU was defined as three months late for a scheduled pharmacy pick‐up appointment. Death and TFO were defined as reported in TIER.Net 20. LTFU status was confirmed by searching for individuals identified as LTFU in the clinic dataset in a province‐wide database that recorded visits to all clinics in the Western Cape. Clients who had transferred to another clinic without informing the clinic staff (silent transfers) and did not have a treatment interruption of more than three months were defined as TFO. Silent transfers who had a treatment interruption of more than three months were documented LTFU. Viral load (VL) suppression was defined as one viral load result < 400 copies/ml.

Baseline characteristics were described using appropriate descriptive statistics. Kaplan‐Meier estimates were used to calculate the cumulative probability of LTFU after starting ART. Secondary analyses in a subset of the study sample that met eligibility criteria for adherence club referral were also completed. Further exploratory analysis that stratified clients by whether they had received clinic or adherence club‐based care were conducted to evaluate LTFU by calendar year of initiating ART. For all time‐to‐event analyses, 14 participants who returned from an adherence club to clinic care during follow‐up were excluded. For the analysis of adherence club referral as a time‐dependent risk factor for LTFU, individual client records were split at the time of adherence club referral.

Univariate and multivariate analyses of LTFU were conducted using Cox proportional‐hazards models. Selection of co‐variates for inclusion in univariate and multivariate modelling was based primarily on clinical relevance and data availability. All available relevant data elements with high quality and completeness were included in the primary and secondary multivariate analyses. Due to high rates of missing data, VL was not included in the models. For the exploratory supplementary modelling stratified by clinic or adherence club care that included smaller sample sizes, a more limited number of baseline data elements of key clinical significance were included. Proportional hazards assumptions were tested using scaled Schoenfeld residuals. Goodness‐of‐fit was assessed using the likelihood‐ratio (LR) test. Referral to an adherence club was not randomly assigned, thus, to account for potential selection bias in the analyses of LTFU, the probability of adherence club referral was calculated using inverse probability weighting and incorporated into Cox proportional hazards models 22, 23. Analyses were performed using Stata Version 14 (StataCorp LP, College Station, TX, USA).

### 2.4 Survey study design, data management and analysis

Data on client experiences of receiving ART adherence club support and healthcare workers perceptions of delivering ART adherence club support were collected through an anonymous self‐administered questionnaire. Convenience sampling was used. Client participants were invited to enrol in the study after they had completed their adherence club visit. Enrolment of healthcare workers also took place at the end of the working day at the clubs. All health workers directly and indirectly involved with the club were invited to take part in the survey.

Survey data were collected between August and September 2017 and all participants signed informed consent. The questionnaire included closed‐ and open‐ended questions in all three of the most commonly spoken languages of the area. For closed‐ended questions, responses were selected from a drop‐down option list and included Likert‐scale grading from strongly disagree to strongly agree. Data were entered directly into an electronic data capture device. Standard observational statistical tests were used to evaluate the frequency of participant responses. Thematic analysis was used to analyse responses to open‐ended questions. All analyses were completed in Stata Version 14 (StataCorp LP, College Station, TX, USA).

### 2.5 Ethics

Ethical approval, including a waiver for informed consent for the cohort analysis of routine data, was obtained from the Stellenbosch University Ethics Committee (Reference number N17/05/056).

## 3 Results

### 3.1 Cohort study results

#### 3.1.1 Baseline

A total of 465 clients were included in the cohort study (Table 2). The majority were women (299, 64.3%). Median baseline CD4 count was 375 (IQR: 234 to 532) cells/μL and median age was 32 (IQR: 27 to 40) years. Women comprised 70.8% of club clients compared with 59.3% of those remaining in clinic care (*p* = 0.010). The median baseline CD4 count was higher among those referred to adherence clubs (399 vs. 345 cells/μL, *p* = 0.004) and fewer referred to adherence clubs had baseline TB (3.5% vs. 12.6%, *p* = 0.002).

**Table 2:** Baseline characteristics

	Clinic n (%)	ART Club n (%)	Total n (%)	*p* value
Study sample				
N	263 (56.6)	202 (43.4)	465 (100)	
Gender				
Female	156 (59.3)	143 (70.8)	299 (64.3)	0.010
Male	107 (40.7)	59 (29.2)	166 (35.7)	
Age (years)				
Median (IQR)	32 (27 to 41)	32 (27 to 40)	32 (27 to 40)	0.919
18 to 25	56 (21.3)	40 (19.8)	96 (20.7)	0.963
26 to 35	108 (41.1)	84 (41.6)	192 (41.3)	
36 to 45	61 (23.2)	46 (22.8)	107 (23)	
>45	38 (14.5)	32 (15.8)	70 (15.1)	
Pregnant at ART start^a^				
Yes	22 (14.1)	25 (17.5)	47 (15.7)	0.155
Baseline CD4 count (cells/μL)				
Median (IQR)	345 (198 to 508)	399 (289 to 539)	375 (234 to 532)	0.004
0 to 200	67 (25.5)	26 (12.9)	93 (20)	0.010
201 to 250	65 (24.7)	49 (24.3)	114 (24.5)	
351 to 500	61 (23.2)	64 (31.7)	125 (26.9)	
>500	68 (25.9)	62 (30.7)	130 (28)	
Missing	2 (0.8)	1 (0.5)	3 (0.7)	
Previous ART exposure				
None	240 (91.3)	177 (87.6)	417 (89.7)	0.077
PMTCT	18 (6.8)	24 (11.9)	42 (9)	
>30 days	5 (1.9)	1 (0.5)	6 (1.3)	
Baseline TB				
None	226 (85.9)	192 (95.1)	418 (89.9)	0.002
Yes	33 (12.6)	7 (3.5)	40 (8.6)	
Missing	4 (1.5)	3 (1.5)	7 (1.5)	
Year of ART start				
2014	125 (47.5)	101 (50)	226 (48.6)	0.597
2015	138 (52.5)	101 (50)	239 (51.4)	

a Denominator for % = number of women in clinic or ART group. For all other factors denominator for % = N.

#### 3.1.2 Entry into adherence club care

In total, 202 (43.4%) and 263 (56.6%) clients were referred to an adherence club or remained in clinic care respectively. Of the 202 clients referred to an adherence club, 21 (10.4%) were referred during the first six months of ART.

#### 3.1.3 Loss to Follow‐Up (LTFU)

**Figure 1:**
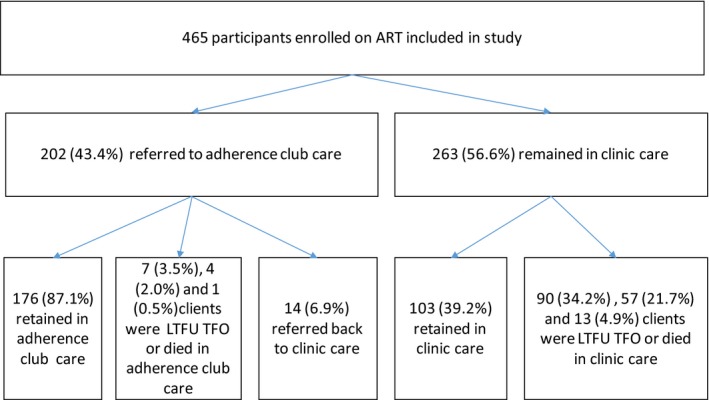
Outline of client outcomes over follow‐up period.

**Figure 2:**
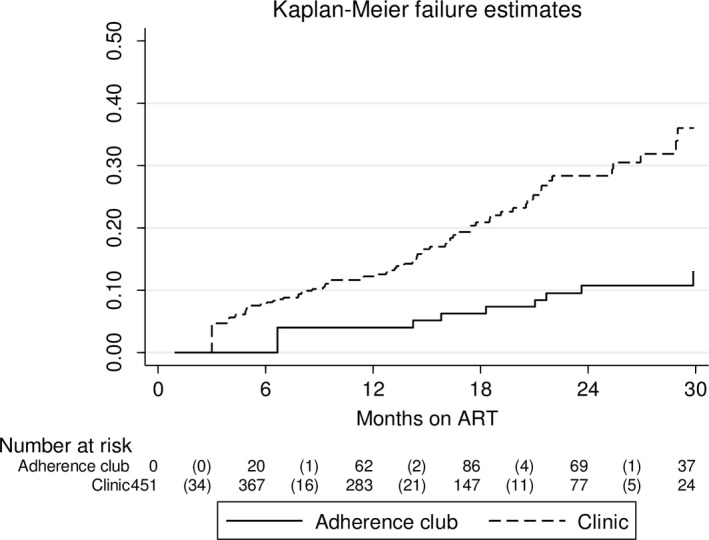
Kaplan‐Meier estimate of UTFU. Restricted to 451 clients Excluded 14 who reverted to clinic after referral to a clinic‐ or community‐based adherence club. Log‐rank test for equality of survivor functions *p* < 0.001.

**Figure 3:**
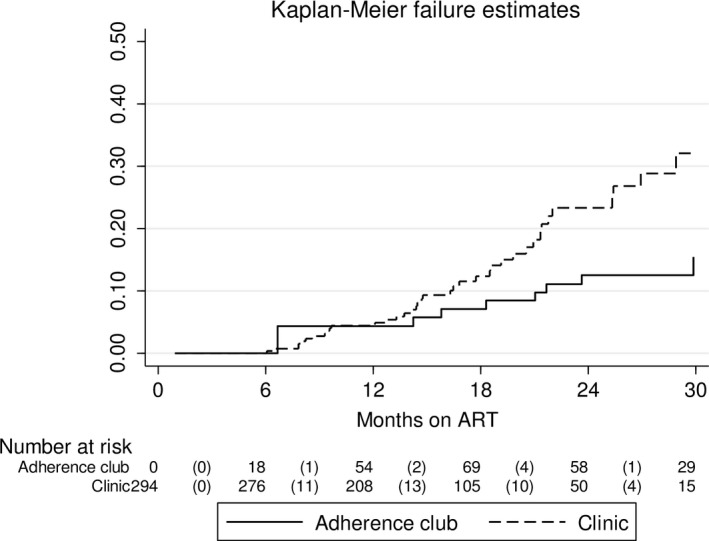
Kaplan‐Meier estimate of LTFU status restricted clients eligible for adherence club referral. Restricted to 294 clients who were eligible for clinic or community‐based adherence club referral and remained in adherence club care after adherence club referral. Log‐rank for equality of survivor functions *p* < 0.001.

Median follow‐up time was 20.7 months (IQR 14.1 to 27.7). The total person years of follow‐up was 653, of which 449 contributed by clients in clinic care and 204 by clients in adherence club care. The total number of clients LTFU, died and TFO from adherence club care was 7 (3.5%), 4 (2.0%) and 1 (0.5%) respectively. The total number of clients LTFU, TFO and died from clinic care was 90 (34.2%), 57 (21.7%) and 13 (4.9%) respectively (Figure 1). Kaplan‐Meier estimates showed LTFU was lower in clients referred to adherence clubs, both overall (*p* < 0.001) (Figure 2) and when restricting analysis to clients eligible for club referral (*p* < 0.001) (Figure 3).

Multivariate analysis confirmed lower LTFU among clients referred to adherence clubs (aHR = 0.25, 95% CI: 0.11 to 0.56) (Table 3). There was higher LTFU among clients starting ART in 2015 compared with 2014 (aHR = 1.60, 95% CI: 1.00 to 2.57). Multivariate analysis restricted to 294 clients eligible for adherence club referral also showed lower LTFU among clients referred to adherence clubs (aHR = 0.28, 95% CI: 0.12 to 0.65) (Table 4). LTFU among clients starting ART in 2015 compared with 2014 was also higher on this restricted analysis (aHR = 2.31, 95% CI: 1.21 to 4.42). No other measured baseline factors were associated with LTFU on multivariate analysis.

**Table 3:** Cox regression of baseline factors and LTFU

	Crude hazard ratio (95% CI)		Adjusted hazard ratio (95% CI)	*p* value
Referred to club				
Yes	0.29 (0.14 to 0.64)	0.002	0.25 (0.11 to 0.56)	0.001
No	1		1	
Gender				
Male	0.95 (0.63 to 1.42)	0.798	1.09 (0.7 to 1.69)	0.704
Female	1		1	
Age category (years)				
15 to 25	1.48 (0.9 to 2.43)	0.067	1.45 (0.86 to 2.45)	0.067
26 to 35	1		1.0	
36 to 45	1.08 (0.64 to 1.83)		1.12 (0.66 to 1.91)	
>45	0.71 (0.34 to 1.46)		0.75 (0.36 to 1.58)	
Baseline CD4 (cells/μL)				
> 500	1	0.008	1.0	0.009
351 to 500	0.71 (0.42 to 1.2)		0.62 (0.36 to 1.05)	
201 to 350	0.69 (0.4 to 1.19)		0.68 (0.39 to 1.19)	
0 to 200	0.69 (0.38 to 1.25)		0.63 (0.33 to 1.19)	
Pregnant at baseline				
Yes	1.69 (0.9 to 3.18)	0.101	1.79 (0.89 to 3.63)	0.103
Baseline TB				
Yes	1.29 (0.69 to 2.42)	0.427	1.41 (0.73 to 2.73)	0.306
Year ART start				
2014	1		1.0	
2015	1.38 (0.89 to 2.13)	0.151	1.60 (1.00 to 2.57)	0.049

This analysis was restricted to 451 clients, excluding 14 who reverted to clinic care after referral to a community‐based adherence club.

**Table 4:** Cox regression of baseline factors and LTFU restricted to patients eligible for adherence club referral

	Crude hazard ratio (95% CI)	*p* value	Adjusted hazard ratio (95% CI)	*p* value
Referred to club				
Yes	0.33 (0.14 to 0.74)	0.007	0.28 (0.12 to 0.65)	0.003
No	1.0		1.0	
Gender				
Male	1.34 (0.76 to 2.37)	0.314	1.4 (0.78 to 2.51)	0.255
Female	1.0		1.0	
Age category (years)				
15 to 25	1.66 (0.85 to 3.25)	0.210	1.79 (0.85 to 3.8)	0.215
26 to 35	1.0		1.0	
36 to 45	0.71 (0.31 to 1.61)		0.65 (0.3 to 1.41)	
>45	0.79 (0.3 to 2.1)		0.84 (0.31 to 2.29)	
Baseline CD4 (cells/μL)				
>500	1.0		1.0	0.354
351 to 500	0.85 (0.4 to 1.8)	0.436	0.73 (0.35 to 1.52)	
201 to 350	0.95 (0.43 to 2.1)		0.94 (0.42 to 2.11)	
0 to 200	1.29 (0.53 to 3.14)		1.42 (0.58 to 3.46)	
Year ART start				
2014	1.0		1.0	0.012
2015	1.95 (1.01 to 3.74)	0.045	2.31 (1.21 to 4.42)	

This analysis was restricted to 292 clients who were eligible for community‐based adherence club referral (on the same ART regimen for >6 months, not pregnant at baseline, no active TB at baseline) and remained in adherence club care after adherence club referral.

Based on exploratory analysis, further multivariate analysis was conducted, which split the data for clients in clinic care and those attending adherence clubs. Analysis on 447 clients in clinic care confirmed increased LTFU among individuals starting ART in 2015 (aHR = 1.76, 95% CI: 1.10 to 2.81) compared with those starting ART in 2014 (Table S1). Analysis on 177 clients in adherence club care, conversely, did not show increased LTFU among clients starting ART in 2015 (aHR = 0.43, 95% CI: 0.04 to 4.32) (Table S2).

##### Viral load

The proportions of clients completing six, twelve and twenty‐four months on ART with a viral load reported between zero and six, seven and twelve, thirteen and twenty‐four months of ART were 40.6%, 36.9% and 62.9% respectively (Table 5). Viral load suppression rates for clients in clinic care were 87.3% (95% CI: 81.0 to 92.0), 91% (95% CI: 83.6 to 95.8) and 76.9% (95% CI: 63.2 to 87.5) during these time intervals. Viral load suppression rates for clients in adherence club care were higher between 0 and 6 months (100%) and 13 and 24 months (97%; 95% CI: 87.7 to 99.9) months and similar between 7 and 12 months of ART (90.3%; 95% CI: 74.2 to 97.9).

**Table 5:** Viral load suppression rates

	0 to 6 Months ART			7 to 12 Months ART			13 to 24 Months ART			Total		
	VLD	VLS (n)	VLS % (95% CI)	VLD	VLS (n)	VLS % (95% CI)	VLD	VLS (n)	VLS % (95% CI)	VLD	VLS (n)	VLS % (95% CI)
Clinic	157	137	87.3 (81.0 to 92.0)	100	91	91.0 (83.6 to 95.8)	52	40	76.9 (63.2 to 87.5)	309	268	86.7 (82.4 to 90.3)
Adherence club	5	5	100	31	28	90.3 (74.2 to 97.9)	43	42	97.0 (87.7 to 99.9)	79	75	94.9 (87.5 to 98.6)
Total	162	142	87.7 (81.6 to 92.3)	131	119	90.8 (85.5 to 95.2)	95	82	86.3 (77.7 to 92.5)	388	343	88.4 (84.8 to 91.4)

VLD, Viral load done and reported; VLS, Viral load suppressed.

### 3.2 Survey data

#### 3.2.1 Client experiences

**Figure 4:**
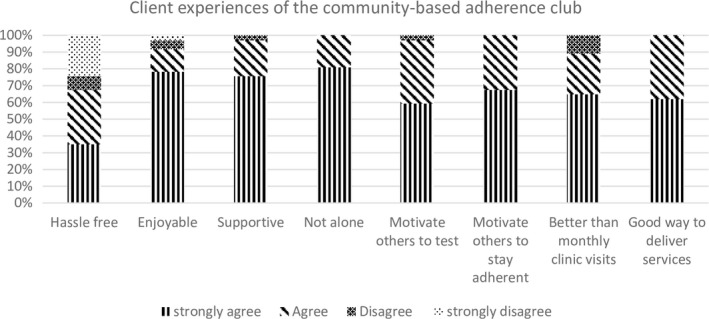
Client experiences of community‐based adherence club (n = 37). Restricted to clients attending a community‐based adherence club.

Overall, 37 participants, 33 (89.2%) women and 4 (10.8%) men with a median age of 33 years completed the client survey (Figure 4 and Table S3a). Twenty‐five participants (68%) had been on ART for ≥ 3 years. More than 50% had been in the adherence club for < 1 year. All survey participants agreed that adherence clubs were a good way to deliver high quality health services for HIV‐positive individuals and that participating in an adherence club empowered them to motivate others to be adherent to ART (Figure 4). The majority of participants agreed that being part of an adherence club was an enjoyable and supportive experience and that attending the adherence club was better than monthly clinic visits. Almost all (36, 97%) participants believed that clients who attend the clinic for ART did not receive a better service than those who received ART from an adherence club. A minority (6, 16%) reported being worried that they may see someone whom they do not trust when they collect their ART at the adherence club. Twelve (32%) participants did not agree that the adherence club was hassle free for them (Table S3d).

#### 3.2.2 Healthcare worker perceptions

**Figure 5:**
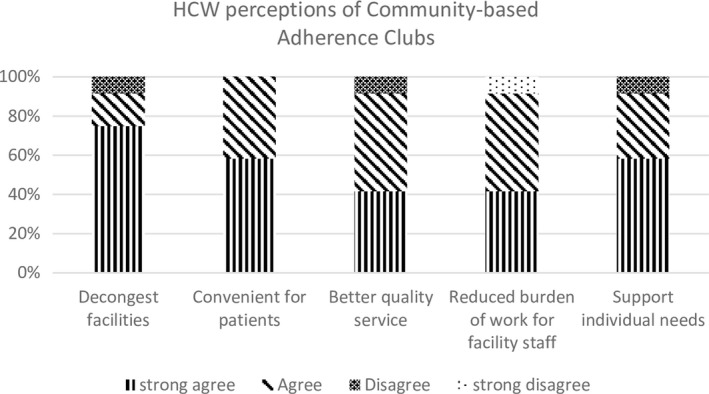
Healthcare worker perceptions of adherence clubs (n = 12).

Overall, 12 healthcare workers (nurses, CHWs, pharmacy workers and co‐ordinators/managers) with a mean age of 49 years completed the healthcare worker survey (Table S4a,b,c). Half were women and the length of time they had worked in the health service was one to ten years. Healthcare workers perceived adherence clubs to be “positive” and the majority agreed that adherence clubs were an effective way to decongest clinics, provided a better quality service for clients than monthly clinic visits, were convenient, reduced the burden on facility‐level personnel, and supported client needs (Figure 5, Table 4c).

#### 3.2.3 Key factors to successful adherence club implementation

The majority of clients and healthcare workers agreed that the ideal number of clients per club should be ≥ 20, counsellors should lead the management of club support, and churches and community halls were the most appropriate venues. Substance abuse and not having disclosed their HIV status to those with whom they live were reported as barriers to successful club attendance. All healthcare workers agreed it was essential to know that a client is stable on ART before transferring them to an adherence club. They reported that clients struggle with adherence when; (i) they have not disclosed their HIV status to anyone, (ii) they feel healthy and do not feel that they need ART and (iii) when they travel to other places.

Communication between clinic staff and those leading the adherence club as well as between adherence club staff and the client was reported as a factor critical for adherence club success. Healthcare workers reported that clients who do not collect their medication and the transportation of medication to the club as major challenges to successful adherence club implementation. Further key considerations for successful club implementation included short waiting times and short travel distance to collect ART (Tables S3 and S4).

## 4 Discussion

Overall, LTFU in this cohort was high (20.9%), similar to that reported for other clinics in the sub‐district during the same period 24. In line with some previous publications 11, 12, 23. LTFU was much lower among clients referred to adherence clubs, when compared to those who stayed in clinic care. Viral load suppression rates ranged from 86.3% to 90.8% and were higher among clients in adherence clubs compared with those who remained in clinic care. The majority of clients referred to adherence clubs, who were not LTFU, were still in the adherence club system at the end of the follow‐up period, with only 14 clients returning to clinic care. The reasons for this were not recorded in this dataset; however, anecdotally it is likely to be because they developed a clinical illness, such as TB, requiring regular care at the clinic.

LTFU was higher among clients starting ART in 2015 compared with 2014. In subset analysis limited to clients in clinic care, this trend was confirmed. However, in subset analysis limited to clients in adherence club care there was no significant difference in LTFU among those starting ART in 2014 and 2015. This suggests that the increased LTFU among clients starting ART in 2015 was driven by those in clinic care, to an extent not fully adjusted for in multivariate analysis. This finding is supported by previously reported higher LTFU in ART clinics, when the numbers of clients on treatment increases 3, 4. By contrast, the data also did not show an increase in LTFU from adherence clubs as the numbers of clients attending the clubs increased. This is a promising finding, although the club system was relatively new and future evaluation of LTFU trends as clubs become busier is needed.

Adherence club guidelines carefully select “stable” clients for adherence club referral 19, and this is likely to have significantly contributed towards the lower LTFU in this group 19. These data do, however, reflect a real world scenario and the results are therefore promising as they suggest the selection of clients for referral to adherence clubs was effective. Furthermore, analysis restricted to clients meeting adherence club referral criteria, confirmed lower LTFU among clients attending adherence clubs.

The client and healthcare worker survey results, in agreement with other studies’ showed high rates of acceptability and support for adherence clubs for ART delivery 5, 25. Clients and healthcare workers both reported that clubs provide an overall positive experience and a better quality ART service compared with visiting the clinic monthly. Clients and healthcare workers were in agreement on key health systems issues for effective club implementation, which largely reinforced issues highlighted in previous publications 26, 27, 28, 29, These included: (i) referral of stable clients to the club, (ii) an ideal club size of ≥20 members (the desired upper limit was not reported), (iii) club services led by a counsellor (iv) using churches or community halls as venues (v) effective communication between all parties, and (vi) timely delivery of prepacked medication.

Previous publications have reported concern over stigma in a club setting 26, and some clients did report fears of being recognized by community members when attending the clubs. In contrast to other published data 15, ART stock‐outs were not reported as a challenge. ART stock‐outs are rare in the Western Cape and it may be that timeous provision of ART is more of a challenge in other settings. In reality what works best when implementing adherence clubs is likely to vary between countries and regions. Publications to date, do, however, consistently refer to many of these same key considerations for effective adherence club implementation, which can be used to develop a set of guiding principles adaptable to different settings.

There is increasing recognition that one size does not fit all for ART care and strong support for differentiated models of care as a strategy to cope with the increasing demands placed on PHC services in high burden settings 5. Further scale‐up of adherence clubs across high burden regions, including expanded access to children and adolescents, can be an effective component of this strategy 30. There are concerns about sustaining the high‐quality clinical outcomes achieved with adherence club systems to date. How best to scale‐up adherence clubs is a subject for debate 27. Effective electronic data monitoring systems that integrate data between clubs and health facilities within the same region can be a critical tool for monitoring and evaluation of adherence club activities. Clubs situated in community venues provide an opportunity to enhance client‐centeredness and increase community buy‐in for health service in line with WHO recommendations 31, 32. A recent study from Malawi reported a reduction in costs when implementing a differentiated model of care that included adherence clubs; however, there remains an urgent need for further data to guide cost effective implementation of adherence club models of care 33.

### 4.1 Strengths and limitations

This cohort study used routine data prospectively strengthened through support provided by PEPFAR implementing partners. The research team was directly involved with adherence club implementation, ensuring relevant interpretation of results. LTFU status was confirmed by reviewing databases that covered clinics from across the Western Cape, to mitigate against clients silently transferring to another clinic and being incorrectly defined as LTFU.

The study has a number of important limitations. As previously mentioned, the non‐random allocation of stable clients for adherence club referral is likely to have significantly contributed towards the lower LTFU in this group. We are not aware of any further key unreported confounders in this study, however, presence of other chronic disease and mental health issues, not measured in this dataset, may have resulted in selection bias in those individuals referred to adherence clubs, which was in turn associated with lower risk of LTFU. This study reported on women pregnant at baseline but did not capture pregnancy occurring on ART; however, there does not appear to be any clear rationale for this to have led to differential bias in reporting of the primary and secondary outcomes. The study clinic was included in a community randomized trial with ART provided for all HIV‐positive clients from 2014, increased ART enrolments and some additional clinical staff. Further to this, all clinic activities were aligned to standard care and we therefore do not believe that this has significantly biased study outcomes nor does it limit generalizability of these results.

The analysis also reports on a small cohort, which may have limited external applicability of results. The survey of clients and healthcare workers was based on rapid, consumer‐satisfaction style measures in a convenience sample of participants; which also limited generalizability of these results. In addition, survey data collection was delayed for logistical reasons until 2017, after completion of cohort follow‐up. Anecdotally circumstances at the adherence clubs did not change significantly between the period of follow‐up for the cohort study and survey completion. We therefore believe that when the survey results are considered, with the background information provided about the study clinic and adherence clubs, they can be of significant use to implementers at other comparable sites, and could furthermore contribute to development of larger future surveys.

## 5 Conclusions

Differentiated models of care that include community‐based adherence clubs are likely to become an increasingly important component of PHC ART delivery. This study adds new data from a rural context to the growing evidence showing promising clinical outcomes and high levels of acceptability of the adherence club model.

## Competing interest

No authors declare a competing interest.

## Authors’ contributions

PB, CG, GH and SM conceptualized the manuscript. All authors contributed towards development of the manuscript and reviewed drafts including the final draft in this submission.

## Supporting information


**Table S1.** Factors associated with loss to follow‐up among clients in clinic care

**Table S2.** Factors associated with loss to follow‐up among clients in adherence clubs

**Table S3.** Tables for Client survey responses (n = 37). (**a**) Demographic data of clients attending Adherence Club (n = 37). (**b**) Client Experiences of ART (n = 37). (**c**) Reasons for treatment interruptions (n = 37). (**d**) Experience of club‐based adherence support (n = 37). (**e**) How to Improve Clubs (n = 37). (**f**) Final words (n = 37)

**Table S4.** Tables for Healthcare worker survey responses (n = 12). (**a**) Demographic data of healthcare workers included in study (n = 12). (**b**) Adherence Support (n = 12). (**c**) Adherence support continued (n = 12). (**d**) How to improve clubs (n = 12). (**e**) Satisfaction with work implementing ART adherence support (n = 12). (**f**) Final words (n = 12)

## References

[1] World Health Organization . Guideline on when to start antiretroviral therapy and on pre‐exposure prophylaxis for HIV. Geneva: World Health Organization; 2015. 26598776

[2] Western Cape Department of Health . The Western Cape Antiretroviral Treatment Guidelines Cape Town Western Cape Department of Health. 2015.

[3] Cornell M , Grimsrud A , Fairall L , Fox MP , van Cutsem G , Giddy J , et al. Temporal changes in programme outcomes among adult patients initiating antiretroviral therapy across South Africa, 2002‐2007. AIDS. 2010;24(14):2263–70. 20683318 10.1097/QAD.0b013e32833d45c5 PMC2948209

[4] Fatti G , Grimwood A , Mothibi E , Shea J . The effect of patient load on antiretroviral treatment programmatic outcomes at primary health care facilities in South Africa: a multicohort study. J Acquir Immune Defic Syndr. 2011;58(1):e17–9. 21860361 10.1097/QAI.0b013e318229baab

[5] Grimsrud A , Bygrave H , Doherty M , Ehrenkranz P , Ellman T , Ferris R , et al. Reimagining HIV service delivery: the role of differentiated care from prevention to suppression. J Int AIDS Soc. 2016;19(1):21484. 27914186 10.7448/IAS.19.1.21484 PMC5136137

[6] Pathmanathan I , Pevzner E , Cavanaugh J , Nelson L . Addressing tuberculosis in differentiated care provision for people living with HIV. Bull World Health Organ. 2017;95(1):3. 28053356 10.2471/BLT.16.187021 PMC5180337

[7] Bemelmans M , Baert S , Goemaere E , Wilkinson L , Vandendyck M , van Cutsem G , et al. Community‐supported models of care for people on HIV treatment in sub‐Saharan Africa. Trop Med Int Health. 2014;19(8):968–77. 24889337 10.1111/tmi.12332

[8] Western Cape Department of Health . The Western Cape Antiretroviral Treatment Guidelines. Cape Town: Western Cape Department of Health; 2013.

[9] Western Cape Department of Health . Guidelines for ART clubs. Cape Town: Western Cape Department of Health; 2015.

[10] Tsondai PR , Wilkinson LS , Grimsrud A , Mdlalo PT , Ullauri A , Boulle A . High rates of retention and viral suppression in the scale‐up of antiretroviral therapy adherence clubs in Cape Town, South Africa. J Int AIDS Soc. 2017;20 Suppl 4:21649. 28770595 10.7448/IAS.20.5.21649 PMC5577696

[11] Luque‐Fernandez MA , Van Cutsem G , Goemaere E , Hilderbrand K , Schomaker M , Mantangana N , et al. Effectiveness of patient adherence groups as a model of care for stable patients on antiretroviral therapy in Khayelitsha, Cape Town, South Africa. PLoS ONE. 2013;8:e56088. 23418518 10.1371/journal.pone.0056088 PMC3571960

[12] Jobarteh K , Shiraishi RW , Malimane I , Samo Gudo P , Decroo T , Auld AF , et al. Community ART support groups in Mozambique: the potential of patients as partners in care. PLoS One. 2016;11:e0166444. 27907084 10.1371/journal.pone.0166444 PMC5132187

[13] Grimsrud A , Sharp J , Kalombo C , Bekker LG , Myer L . Implementation of community‐based adherence clubs for stable antiretroviral therapy patients in Cape Town, South Africa. J Int AIDS Soc. 2015;18:19984. 26022654 10.7448/IAS.18.1.19984 PMC4444752

[14] Hanrahan CF , Schwartz SR , Mudavanhu M , West NS , Mutunga L , Keyser V , et al. The impact of community‐ versus clinic‐based adherence clubs on loss from care and viral suppression for antiretroviral therapy patients: findings from a pragmatic randomized controlled trial in South Africa. PLoS Med. 2019;16:e1002808. 31112543 10.1371/journal.pmed.1002808 PMC6528966

[15] Tshuma N , Mosikare O , Yun JA , Alaba OA , Maheedhariah MS , Muloongo K , et al. Acceptability of community‐based adherence clubs among health facility staff in South Africa: a qualitative study. Patient Prefer Adherence. 2017;11:1523–31. 28979100 10.2147/PPA.S116826 PMC5602677

[16] Horwood CM , Youngleson MS , Moses E , Stern AF , Barker PM . Using adapted quality‐improvement approaches to strengthen community‐based health systems and improve care in high HIV‐burden sub‐Saharan African countries. AIDS. 2015;29 Suppl 2:S155–64. 26102626 10.1097/QAD.0000000000000716

[17] Hayes R , Ayles H , Beyers N , Sabapathy K , Floyd S , Shanaube K , et al. HPTN 071 (PopART): rationale and design of a cluster‐randomised trial of the population impact of an HIV combination prevention intervention including universal testing and treatment ‐ a study protocol for a cluster randomised trial. Trials. 2014;15(1):57. 24524229 10.1186/1745-6215-15-57 PMC3929317

[18] Western Cape Department of Health . The Western Cape Consolidated Guidelines for HIV Treatment: Prevention of Mother‐ to‐ Child Transmission of HIV (PMTCT), Children, Adolescents and Adults. Cape Town: Western Cape Department of Health; 2016.

[19] Cape Winelands District Department of Health . Guideline for the distribution of pre‐packed medication to stable clients on chronic disease medication at “Fast‐Lane” or “Club” Worcester Cape Winelands District Department of Health. 2016.

[20] Osler M , Hilderbrand K , Hennessey C , Arendse J , Goemaere E , Ford N , et al. A three‐tier framework for monitoring antiretroviral therapy in high HIV burden settings. J Int AIDS Soc. 2014;17:18908. 24780511 10.7448/IAS.17.1.18908 PMC4005043

[21] South African National Department of Health . The electronic TB register (ETR.net) Pretoria. South African National Department of Health. 2017.

[22] Buchanan AL , Hudgens MG , Cole SR , Lau B , Adimora AA ; Women's Interagency HIVS . Worth the weight: using inverse probability weighted Cox models in AIDS research. AIDS Res Hum Retroviruses. 2014;30(12):1170–7. 25183195 10.1089/aid.2014.0037 PMC4250953

[23] Grimsrud A , Lesosky M , Kalombo C , Bekker LG , Myer L . Implementation and operational research: community‐based adherence clubs for the management of stable antiretroviral therapy patients in Cape Town, South Africa: a cohort study. J Acquir Immune Defic Syndr. 2016;71(1):e16–23. 26473798 10.1097/QAI.0000000000000863

[24] Western Cape Department of Health . Western Cape Province HAST Directorate: routine data report. Cape Town Western Cape Department of Health. 2016.

[25] De Jager GA , Crowley T , Esterhuizen TM . Patient satisfaction and treatment adherence of stable human immunodeficiency virus‐positive patients in antiretroviral adherence clubs and clinics. Afr J Prim Health Care Fam Med. 2018;10(1):e1–8. 10.4102/phcfm.v10i1.1759 PMC6018455 29943608

[26] Grimsrud A , Balkan S , Casas EC , Lujan J , Van Cutsem G , Poulet E , et al. Outcomes of antiretroviral therapy over a 10‐year period of expansion: a multicohort analysis of African and Asian HIV programs. J Acquir Immune Defic Syndr. 2014;67(2):e55–66. 24977472 10.1097/QAI.0000000000000268

[27] MacGregor H , McKenzie A , Jacobs T , Ullauri A . Scaling up ART adherence clubs in the public sector health system in the Western Cape, South Africa: a study of the institutionalisation of a pilot innovation. Global Health. 2018;14(1):40. 29695268 10.1186/s12992-018-0351-z PMC5918532

[28] Mukumbang FC , Orth Z , van Wyk B . What do the implementation outcome variables tell us about the scaling‐up of the antiretroviral treatment adherence clubs in South Africa? A document review. Health Res Policy Syst. 2019;17(1):28. 30871565 10.1186/s12961-019-0428-z PMC6419395

[29] Flamig K , Decroo T , van den Borne B , van de Pas R . ART adherence clubs in the Western Cape of South Africa: what does the sustainability framework tell us? A scoping literature review. J Int AIDS Soc. 2019;22:e25235. 30891928 10.1002/jia2.25235 PMC6531844

[30] World Health Organization . Key considerations for differentiated antiretroviral therapy for specific populations. Geneva: World Health Organization 2017.

[31] Rasschaert F , Decroo T , Remartinez D , Telfer B , Lessitala F , Biot M , et al. Sustainability of a community‐based anti‐retroviral care delivery model ‐ a qualitative research study in Tete, Mozambique. J Int AIDS Soc. 2014;17:18910. 25292158 10.7448/IAS.17.1.18910 PMC4189018

[32] World Health Organization . WHO global strategy on people‐centred and integrated health services. Geneva: World Health Organization; 2015.

[33] Prust ML , Banda CK , Nyirenda R , Chimbwandira F , Kalua T , Jahn A , et al. Multi‐month prescriptions, fast‐track refills, and community ART groups: results from a process evaluation in Malawi on using differentiated models of care to achieve national HIV treatment goals. J Int AIDS Soc. 2017;20 Suppl 4:21650. 28770594 10.7448/IAS.20.5.21650 PMC5577715

