# Supplementary material for: Retention in care and factors critical for effectively implementing antiretroviral adherence clubs in a rural district in South Africa
Source: J Int AIDS Soc. 2019 Oct 6;22(10):e25396. doi: 10.1002/jia2.25396 (PMC6778813; doi:10.1002/jia2.25396)
Supplement: Supplementary file 1 — Table S1. Factors associated with loss to follow‐up among clients in clinic care [file JIA2-22-e25396-s001.docx]

**Supplementary material: Table 1:** Factors associated with loss to follow up amongst clients in clinic care

|  |  | **Crude hazard ratio (95% CI)** | **P value** | **Adjusted hazard ratio (95% CI)** | **P value** |
| --- | --- | --- | --- | --- | --- |
| **Gender** | **Male** | 0,96  (0,63-1,45) | 0,840 | 1,06  (0,69-1,61) | 0,792 |
|  | **Female** | 1(-) |  | 1 |  |
| **Age** | **Years** | 0,99  (0,96-1,01) | 0,208 | 0,99  (0,97-1,01) | 0,282 |
| **Baseline CD4**  **(cells/µL)** | **> 500** | (-) | 0,002 | 1(-) | 0,005 |
|  | **350 -500** | 0,64  (0,37-1,11) |  | 0,6  (0,35-1,04) |  |
|  | **200-350** | 0,66  (0,38-1,15) |  | 0,66  (0,37-1,15) |  |
|  | **0-200** | 0,56  (0,3-1,05) |  | 0,58  (0,31-1,07) |  |
| **Year ART start** | **2014** | 1 | 0,023 | 1 | 0,018 |
|  | **2015** | 1,73  (1,08-2,76) |  | 1,76  (1,10-2,81) |  |

This analysis was restricted to person time contributed by 447 individuals in clinics. Baseline covariates including PMTCT status, baseline TB status and ART experience, were excluded from analysis due to collinearity
