# Supplementary material for: Retention in care and factors critical for effectively implementing antiretroviral adherence clubs in a rural district in South Africa
Source: J Int AIDS Soc. 2019 Oct 6;22(10):e25396. doi: 10.1002/jia2.25396 (PMC6778813; doi:10.1002/jia2.25396)
Supplement: Supplementary file 3 — Table S3. Tables for Client survey responses (n = 37). (a) Demographic data of clients attending Adherence Club (n = 37). (b) Client Experiences of ART (n = 37). (c) Reasons for treatment interruptions (n = 37). (d) Experience of club‐based adherence support (n = 37). (e) How to Improve Clubs (n = 37). (f) Final words (n = 37) [file JIA2-22-e25396-s003.docx]

**Supplementary Material 3**

**Supplementary Data Tables for Client survey responses (n=37)**

This supplementary data shows client responses to the study survey for client experiences of community-based adherence club care. Table 1a shows demographic data for this cohort. Table 1b to 1f shows the survey questions and the participant responses to each of these questions. Tables that display frequencies, display data from highest to lowest frequency and not necessarily in the order displayed in the survey.

**Table 3a: Demographic data of clients attending Adherence Club (n=37)**

| **Sex n (%)** |  |  |
| --- | --- | --- |
| male | 4 | 10,81 |
| female | 33 | 89,19 |
|  |  |  |
| **Median age** | 33 (IQR: 29-44) | |
| **Age groups n (%)** |  |  |
| 18-24 | 2 | 5,41 |
| 25-30 | 10 | 27,03 |
| 31-40 | 15 | 40,54 |
| 41-50 | 6 | 16,22 |
| >50 | 4 | 10,81 |
|  |  |  |
| **Time on ART n (%)** |  |  |
| 1-2 yrs | 12 | 32,43 |
| 3-5 yrs | 13 | 35,14 |
| 6-8 yrs | 9 | 24,32 |
| 9-11 yrs | 3 | 8,11 |
| 11-16 yrs | 0 | 0 |
|  |  |  |
| **Yrs in club n (%)** |  |  |
| < 1 yr | 21 | 56.76 |
| 1-2 yrs | 7 | 18,92 |
| 3-5 yrs | 9 | 24,32 |
|  |  |  |

**Table 3b: Client Experiences of ART (n=37)**

|  | **Strongly Agree** | **Agree** | **Disagree** | **Strongly Disagree** | **TOTAL** |
| --- | --- | --- | --- | --- | --- |
| 1. Since I have taking ARVs I have felt my health has improved dramatically | 33 | 4 | 0 | 0 | 37 |
| 2. I find the side effects of taking ARVs difficult to manage/cope with | 3 | 6 | 15 | 13 | 37 |
| 3. It is easy for me to remember to take my ARVs every day | 22 | 8 | 1 | 6 | 37 |
| 4. I hide my ARVs (pills) to prevent people from finding out that I am living with HIV | 3 | 5 | 7 | 22 | 37 |
| 5. I have reliable people in my life whom I can ask to collect my ARVs if I am unavailable myself | 21 | 9 | 4 | 3 | 37 |
| 6. I move around a lot so it is challenging for me to keep to my ART appointments each and every time | 1 | 1 | 20 | 15 | 37 |

**Table 3c: Reasons for treatment interruptions (n=37)**

What are the three most important reasons why patients experience treatment interruptions? (Select 3)

**Frequency**

| Not disclosing their HIV status to people who they share their homes with | 21 |
| --- | --- |
| They are heavy alcohol/drug users and forget to adhere and to keep adherence support appointments | 14 |
| The side effects of ART are too difficult to manage | 11 |
| They have not accepted their HIV+ diagnosis | 10 |
| Not having adequate food to take ART with | 9 |
| Feeling healthier and no longer believing that they need to take the ART | 9 |
| The waiting times at the health facility are too long | 7 |
| They are travelling to other parts of the country (like the Eastern Cape) | 6 |
| They have heard rumours/guess that health staff will treat them badly if they admit to non-adherence | 6 |
| They move around a lot and are often not close to the health facility on their appointment days | 5 |
| The cost of collecting ART is too high | 5 |
| They experience frequent family disturbances/social disputes that make it hard to keep up continuity in their lives | 4 |
| They want a break from daily chronic medication | 1 |
| They have experienced reprimands by health staff about non-adherence and delay disclosing treatment interruptions | 0 |
| They have an alternative health beliefs and do not trust that ARVs are the best treatment for HIV | 0 |
| Their health is not their primary concern at the moment | 0 |
| Another reason not mentioned here | 2* |
| ** These 2 participants did not specify what other important reasons patients experience treatment interruptions. (The survey included free text for other reasons)* |  |

**Table 3d: Experience of club-based adherence support (n=37)**

|  | **Strongly Agree** | **Agree** | **Disagree** | **Strongly Disagree** | **TOTAL** |
| --- | --- | --- | --- | --- | --- |
| 1. Being part of an adherence support club reduces the hassle of being on ART | 13 | 12 | 3 | 9 | 37 |
| 2. Being part of an adherence support club is something I enjoy | 29 | 5 | 2 | 1 | 37 |
| 3. Being part of an adherence support club offers me meaningful social support and counselling | 28 | 8 | 1 | 0 | 37 |
| 4. Being part of an adherence support club reminds me that I am not alone in living with HIV | 30 | 7 | 0 | 0 | 37 |
| 5. I am very worried that someone who I do not trust might see me when I collect my ARVs at the club | 4 | 2 | 6 | 25 | 37 |
| 6. Being part of an adherence support club empowers me to motivate others in my social life to test for HIV and get support | 22 | 14 | 1 | 0 | 37 |
| 7. Being part of an adherence support club empowers me to motivate others, living with HIV to stay adherent/keep taking their ARVs | 25 | 12 | 0 | 0 | 37 |
| 8. Patients who are able to see their counsellor and nurse at the clinic every month receive better overall care than people in clubs | 0 | 1 | 24 | 12 | 37 |
| 9. Being part of an adherence support club is better than having to collect my ART and see the nurse at the clinic every month | 24 | 9 | 4 | 0 | 37 |
| 10. Overall, adherence support clubs are a good way to deliver high quality health services for people living with HIV | 23 | 14 | 0 | 0 | 37 |

**Table 3e:** **How to Improve Clubs (n=37)**

|  |  | |
| --- | --- | --- |
| 1. What sort of things should the health worker worry about before transferring clients to an adherence support club? (Free text) | **Client Responses** | |
| The client is adherent to their treatment | 5 | |
| Distance (presumably travel distance to clinic) | 3 | |
| The client is up to date with treatment | 2 | |
| The client not collecting their medication | 2 | |
| Viral load | 1 | |
| Nothing if they trust the client | 1 | |
| Nothing reported | 23 | |
| TOTAL | 37 | |
|  |  | |
| 2. What is the most appropriate number of patients per club to maximise the quality of adherence support provided to them? | **Frequency** | |
| >24 | 19 |  |
| 20-24 | 6 |  |
| 15-19 | 6 |  |
| 10-14 | 5 |  |
| <10 | 1 |  |
| TOTAL | 37 |  |
| 3. What sort of health worker should lead the management of adherence support offered through the alternative distribution platform? |  | |
|  | **Frequency** |  |
| Counselor | 26 |  |
| PLWHIV who receives training | 4 |  |
| Pharmacist | 4 |  |
| Nurse | 3 |  |
| TOTAL | 37 |  |
|  |  |  |
| 4. What sort of venues are appropriate/good places to host alternative distribution points for adherence support clubs? [please tick all that apply] | **Frequency** | |
|  |  | |
| Church | 19 |  |
| Community Hall | 12 |  |
| Room at facility | 11 |  |
| Member Home | 8 |  |
| Creche | 5 |  |
| School | 1 |  |
| Tavern | 0 |  |
| Alternative Healer | 0 |  |
| Local shops | 0 |  |
| Another place not mentioned here | 4* |  |
| ** 3 participants suggested home deliveries and 1 suggested a library* |  |  |

| 5. What are the three main challenges to delivering successful alternative distribution that you would like policy makers to be aware of? |  |
| --- | --- |
|  | **Client Responses** |
| Communication | 3 |
| Confidentiality | 1 |
| Nothing reported | 33 |
| TOTAL | 37 |
|  |  |
| 6. What are the three main principles for delivering successful alternative distribution that you would like to share with health facility staff? |  |
|  | **Client Responses** |
| Communication | 3 |
| Door to door delivery (to avoid being seen) | 1 |
| Everything is fine | 1 |
| Nothing reported | 32 |
| TOTAL | 37 |
|  |  |
| **Table 3f: Final words (n=37)** |  |
| Is there anything else that you believe we should know as lessons learnt about the challenges and ways to improve adherence support through alternative distribution/clubs? (free text) | **Client Responses** |
| Clubs are less time consuming | 7 |
| Health workers are caring | 1 |
| Clubs allow me to motivate others to test and start ART | 1 |
| Door to door | 1 |
| Nothing reported | 27 |
| TOTAL | 37 |
