# Supplementary material for: Retention in care and factors critical for effectively implementing antiretroviral adherence clubs in a rural district in South Africa
Source: J Int AIDS Soc. 2019 Oct 6;22(10):e25396. doi: 10.1002/jia2.25396 (PMC6778813; doi:10.1002/jia2.25396)
Supplement: Supplementary file 4 — Table S4. Tables for Healthcare worker survey responses (n = 12). (a) Demographic data of healthcare workers included in study (n = 12). (b) Adherence Support (n = 12). (c) Adherence support continued (n = 12). (d) How to improve clubs (n = 12). (e) Satisfaction with work implementing ART adherence support (n = 12). (f) Final words (n = 12) [file JIA2-22-e25396-s004.docx]

**Supplementary Material 4**

**Supplementary Data Tables for Healthcare worker survey responses (n=12)**

This supplementary data shows healthcare worker responses to the study survey for healthcare worker perceptions of community-based adherence clubs. Table 2a shows demographic data for this cohort. Table 2b to 2f shows the survey questions and the healthcare worker responses to each of these questions. Tables that display frequencies, display data from highest to lowest frequency and not necessarily in the order displayed in the survey.

**Table 4a: Demographic data of healthcare workers included in study (n=12)**

| **Sex (n)** |  |  |
| --- | --- | --- |
| male | 6 |  |
| female | 6 |  |
| **Age (n)** |  |  |
| Mean (CI) | 48.8 (29-61) | |
| 18-24 | 0 |  |
| 25-30 | 1 |  |
| 31-40 | 1 |  |
| 41-50 | 3 |  |
| >50 | 7 |  |
|  |  |  |
| **Years worked at facility (n)** |  |  |
| < 2 yrs | 1 |  |
| 2-3 yrs | 1 |  |
| 4-5 yrs | 2 |  |
| 6-7 yrs | 2 |  |
| 8-9 yrs | 4 |  |
| 10 yrs | 2 |  |
|  |  |  |
| **Role in club (n)** |  |  |
| Counselling | 2 |  |
| Logistics | 0 |  |
| Packaging | 2 |  |
| Clinical monitoring | 1 |  |
| Other | 7* |  |

*The survey did not allow the HCW to provide details. This would have included HCW’s playing an indirect role in the club e.g. management at sub-district level

| **Table 4b: Adherence Support (n=12)** |  |
| --- | --- |
| 1. What are the three most important reasons why patients experience treatment interruptions? (Select up to 3) | **Frequency** |
| They have not disclosed their HIV status to people who they share their homes with | 5 |
| They feel healthier and no longer believe that they need to take the ART | 5 |
| They are travelling to other parts of the country (like the Eastern Cape) | 3 |
| The waiting times at the health facility are too long | 2 |
| They move around a lot and are often not close to the health facility on their appointment days | 2 |
| They are heavy alcohol/drug users and forget to adhere and to keep adherence support appointments | 2 |
| They experience frequent family disturbances/social disputes that make it hard to keep up continuity in their lives | 2 |
| The side effects of ART are too difficult to manage | 1 |
| They have heard rumours/guess that health staff will treat them badly if they admit to non-adherence | 1 |
| They do not have adequate food to take ART with | 0 |
| They want a break from daily chronic medication | 0 |
| They have experienced reprimands by health staff about non-adherence and delay disclosing treatment interruptions | 0 |
| The cost of collecting ART is too high | 0 |
| They have not accepted their HIV+ diagnosis | 0 |
| They have an alternative health beliefs and do not trust that ARVs are the best treatment for HIV | 0 |
| Their health is not their primary concern at the moment | 0 |
| Another reason not mentioned here | 0 |

**Table 4c: Adherence support continued (n=12)**

|  | **Strongly Agree** | **Agree** | **Disagree** | **Strongly Disagree** | **TOTAL** |
| --- | --- | --- | --- | --- | --- |
| 2. Alternative distribution of ART through 'adherence clubs' is an effective way to decongest health facilities | 9 | 2 | 1 | 0 | 12 |
| 3. Alternative distribution of ART through “adherence clubs” is more convenient for patients. | 7 | 5 | 0 | 0 | 12 |
| 4. Alternative distribution of ART through “adherence clubs” is a better quality of health service for patients than requiring monthly visits to the health facility. | 5 | 6 | 1 | 0 | 12 |
| 5. Offering alternative distribution of ART through “adherence clubs” reduces the burden of work for health facility staff overall. | 5 | 6 | 0 | 1 | 12 |
| 6. Offering alternative distribution of ART through “adherence clubs” empowers healthcare workers to tailor their adherence support to individual patient needs. | 2 | 9 | 1 | 0 | 12 |

**Table 4d: How to improve clubs (n=12)**

|  | **Strongly Agree** | **Agree** | **Disagree** | **Strongly Disagree** | **Total** |
| --- | --- | --- | --- | --- | --- |
| 1. It is essential to know that a patient is stable before they can be transferred to an alternative distribution adherence support club | 11 | 1 | 0 | 0 | 12 |

| 2. What sort of things should the health worker worry about before transferring clients to a adherence support club? | **HCW Responses** |
| --- | --- |
| Ensure the patient is adherent to ART/viral load undetectable | 4 |
| Ensure the patient is stable on ARVs | 2 |
| Screen for co-infections/pregnancy | 2 |
| Ensure patient wants to be enrolled in adherence club | 2 |
| Ensure patient understands their condition/medication | 2 |
| Ensure patient will take responsibility/lifelong commitment to ART | 1 |
| Ensure patients understands why they are being transferred to an adherence club | 1 |
| Sustainability of ART provision | 1 |
| Potential stigma for patient because their medication is prepacked | 1 |
|  |  |
| 3. What is the most appropriate number of patients per club to maximise the quality of adherence support provided to them? | **Frequency** |
| >24 | 6 |
| 20-24 | 3 |
| 15-19 | 3 |
| 10-14 | 0 |
| <10 | 0 |
| TOTAL | 12 |
|  |  |
| 4. What sort of health worker should lead the management of adherence support offered through the alternative distribution platform? | **Frequency** |
| Counselor | 6 |
| Nurse | 3 |
| Pharmacist | 2 |
| PLWHIV who receives training | 1 |
| TOTAL | 12 |
| 5. What sort of venues are appropriate/good places to host alternative distribution points for adherence support clubs? [please tick all that apply] | **Frequency** |
| Community Hall | 12 |
| Church | 9 |
| School | 4 |
| Member Home | 4 |
| Local shops | 4 |
| Crèche | 3 |
| Alternative Healer | 3 |
| Room at facility | 2 |
| Tavern | 2 |
| Another place not mentioned here | 2* |
| ** Other places reported included library, central distribution site, patients home, mobile services, sports club, NGO, ward based services and buildings close to the clinic* |  |
|  |  |
|  |  |
| 6. What are the three main challenges to delivering successful alternative distribution that you would like policy makers to be aware of? | **HCW Responses** |
| Teams that do not work together | 3 |
| Patients not collecting medication | 3 |
| Patients not being counselled before being transferred to a club | 2 |
| Stakeholders do not understand each others' roles | 1 |
| Processes that are not well coordinated | 1 |
| Staffing | 1 |
| Patients not stable or adherent | 1 |
| Patients 'hopping' between facilities | 1 |
| Safety of patients | 1 |
| Need to be within walking distance for patient | 1 |
| Packaging of medication | 1 |
| Sustainable drug supply | 1 |
| Valid scripts | 1 |
| Data | 1 |
| Confidentiality/privacy | 1 |
| Trustworthy provider | 1 |
| Nothing to report | 1 |
|  |  |
| 7. What are the three main principles for delivering successful alternative distribution that you would like to share with health facility staff? | **HCW Responses** |
| All stakeholders understand their role/commitment | 3 |
| Well trained staff | 2 |
| Collect correct data to improve services | 2 |
| Ensure a safe space for the patient | 2 |
| Patients must understand the system | 2 |
| Empathy and respect for patients | 2 |
| Teamwork | 1 |
| Ensure medication is available | 1 |
| Good clinical governance | 1 |
| Equal health service | 1 |

**Table 4e: Satisfaction with work implementing ART adherence support (n=12)**

|  | **Strongly Agree** | **Agree** | **Disagree** | **Strongly Disagree** | **TOTAL** |
| --- | --- | --- | --- | --- | --- |
| 1. I believe that alternative distribution points for adherence support clubs are an important part of improving the quality of the work that I do for patients. | 7 | 5 | 0 | 0 | 12 |
| 2. I am happy that my patients value the work that I do to deliver ART adherence support linked to the alternative distribution platform. | 5 | 7 | 0 | 0 | 12 |
| 3. I believe that the policy-makers who advocate for alternative distribution as part of the ART adherence support programme know how difficult it is to implement this programme successfully in the real world. | 4 | 3 | 3 | 1 | 11* |

*Missing data for 1 HCW

**Table 4f: Final words (n=12)**

| Is there anything else that you believe we should know as lessons learnt about the challenges and ways to improve adherence support through alternative distribution/clubs? (free text) | **Patient Responses** |
| --- | --- |
| Good communication between stakeholders is important | 3 |
| Adherence clubs are labour intensive | 2 |
| Teamwork is important | 2 |
| The process needs to be managed well | 1 |
| Roving teams are an innovative way to support adherence club implementation | 1 |
| Need to encourage patients to collect medication | 1 |
| Patients must take responsibility for their treatment | 1 |
| Need 'after hours' clubs | 1 |
| Need to upgrade the health system | 1 |
| Patient compliance and non-collection of medication should be discussed | 1 |
| Need integrated clubs for all chronic patients | 1 |
| Need to analyses data to improve services | 1 |
